# Haplotypes of the D-Amino Acid Oxidase Gene Are Significantly Associated with Schizophrenia and Its Neurocognitive Deficits

**DOI:** 10.1371/journal.pone.0150435

**Published:** 2016-03-17

**Authors:** Yu-Li Liu, Sheng-Chang Wang, Hai-Gwo Hwu, Cathy Shen-Jang Fann, Ueng-Cheng Yang, Wei-Chih Yang, Pei-Chun Hsu, Chien-Ching Chang, Chun-Chiang Wen, Jyy-Jih Tsai-Wu, Tzung-Jeng Hwang, Ming H. Hsieh, Chen-Chung Liu, Yi-Ling Chien, Chiu-Ping Fang, Stephen V. Faraone, Ming T. Tsuang, Wei J. Chen, Chih-Min Liu

**Affiliations:** 1 Center for Neuropsychiatric Research, National Health Research Institutes, Miaoli 35053, Taiwan; 2 Department of Psychiatry, National Taiwan University Hospital and National Taiwan University College of Medicine, Taipei 10051, Taiwan; 3 Graduate Institute of Brain and Mind Sciences, College of Medicine, National Taiwan University, Taipei 10051, Taiwan; 4 Institute of Biomedical Sciences, Academia Sinica, Taipei 11529, Taiwan; 5 Institute of Bioinformatics, National Yang-Ming University, Taipei 112, Taiwan; 6 Graduate Institute of Biomedical Electronics and Bioinformatics, National Taiwan University, Taipei 10051, Taiwan; 7 Department of Medical Research, National Taiwan University Hospital, Taipei 10051, Taiwan; 8 Medical Genetics Research Center and Department of Psychiatry and Neuroscience & Physiology, SUNY Upstate Medical University, Syracuse, NY 13210, United States of America; 9 Harvard Institute of Psychiatric Epidemiology and Genetics, and Departments of Epidemiology and Psychiatry, Harvard University, Boston, Massachusetts, 02115, United States of America; 10 Institute of Behavioral Genomics, University of California San Diego, San Diego, California 92093, United States of America; 11 Institute of Epidemiology and Preventive Medicine, College of Public Health, National Taiwan University, Taipei 10051, Taiwan; Kunming Institute of Zoology, Chinese Academy of Sciences, CHINA

## Abstract

D-amino acid oxidase (DAO) has been reported to be associated with schizophrenia. This study aimed to search for genetic variants associated with this gene. The genomic regions of all exons, highly conserved regions of introns, and promoters of this gene were sequenced. Potentially meaningful single-nucleotide polymorphisms (SNPs) obtained from direct sequencing were selected for genotyping in 600 controls and 912 patients with schizophrenia and in a replicated sample consisting of 388 patients with schizophrenia. Genetic associations were examined using single-locus and haplotype association analyses. In single-locus analyses, the frequency of the C allele of a novel SNP rs55944529 located at intron 8 was found to be significantly higher in the original large patient sample (p = 0.016). This allele was associated with a higher level of DAO mRNA expression in the Epstein-Barr virus-transformed lymphocytes. The haplotype distribution of a haplotype block composed of rs11114083-rs2070586-rs2070587-rs55944529 across intron 1 and intron 8 was significantly different between the patients and controls and the haplotype frequencies of AAGC were significantly higher in patients, in both the original (corrected p < 0.0001) and replicated samples (corrected p = 0.0003). The CGTC haplotype was specifically associated with the subgroup with deficits in sustained attention and executive function and the AAGC haplotype was associated with the subgroup without such deficits. The DAO gene was a susceptibility gene for schizophrenia and the genomic region between intron 1 and intron 8 may harbor functional genetic variants, which may influence the mRNA expression of DAO and neurocognitive functions in schizophrenia.

## Introduction

D-amino acid oxidase (DAO) has been found to be involved in the signal transduction pathway of the N-methyl-D-aspartic acid (NMDA) receptor [1] and has been hypothesized to be implicated in the pathogenesis of schizophrenia. DAO [2, 3] and DAO activator (DAOA) [4] have been suggested as candidate genes for schizophrenia. The single nucleotide polymorphisms (SNPs) found most consistently to have a significant association with schizophrenia included DAO-M4 (rs2111902 at intron 1), DAO-M5 (rs3918346 at intron 3), and DAO-M6 (rs3741775 at intron 4) in French-Canadian, Russian [4], German [5] and Irish populations [6]; however, these results were not confirmed in a Chinese population [7], and in our previous study using a Taiwanese sample [8].

The use of endophenotypes to refine the phenotypic characterization of schizophrenia has been advocated [9]. There is substantial empirical evidence to support both sustained attention impairments and executive dysfunction as endophenotypic markers for schizophrenia. Sustained attention deficit measured by the Continuous Performance Test (CPT) [10] has been shown to be present in patients with schizophrenia as well as subjects with schizotypal personality disorder and in nonpsychotic relatives of schizophrenic patients [11–17]. Schizophrenic patients [18, 19] and their first degree relatives [20] often have impaired executive function, as measured by the Wisconsin Card Sorting Test (WCST) [21]. Using performance on the CPT and the WCST to define endophenotypes of both impaired sustained attention and executive function in schizophrenia might help address the issue of heterogeneity in schizophrenia by analysis of the association.

As the haplotype structure of the DAO genomic region is different in Caucasian and Asian populations, the associated genomic region may be different in different ethnic samples. Therefore, our aim was to re-sequence the genomic regions of this gene in our sample, and to study the associations between the specific SNPs or haplotypes found and the subgroups of schizophrenia as assessed by CPT and WCST.

## Materials and Methods

### Subjects

#### Subjects for direct sequencing

Study subjects included the probands from the Study on Etiological Factors of Schizophrenia, in which schizophrenic probands from both simplex (i.e. without affected siblings) and multiplex families (i.e. at least two affected siblings) were recruited from National Taiwan University Hospital (Taipei, Taiwan) and Ju-Shan Psychiatric Hospital (Tao-Yuan County, Taiwan) from 2002 to 2005. The exclusion criteria were: severe neurological abnormality, prominent substance abuse, mental retardation, and aboriginal Taiwanese ancestry. Healthy persons with no history of psychiatric disorders and no family history of schizophrenia in their first degree relatives were recruited as controls. A total of 50 independent multiplex patients, 50 simplex patients, and 50 normal controls were selected for the direct sequencing study.

#### Subjects for genotyping

The patients were the affected siblings of two types of families: the multiplex families and the simplex families. The multiplex families were recruited from two research programs: the Multidimensional Psychopathology Study of Schizophrenia [22] from 1993 to 2001; and the Taiwan Schizophrenia Linkage Study [23, 24] from 1998 to 2002. The 93 families from the first research program were interviewed by research psychiatrists and assessed using the Psychiatric Diagnostic Assessment [25]. The 609 families from the second research program were interviewed by well-trained research assistants using the Diagnostic Interview for Genetic Studies [26]. For both studies, the final research diagnosis was formulated by integrating the data from both interviews and clinical information from medical records, based upon the criteria of the Diagnostic and Statistical Manual of Mental Disorders, 4^th^ edition (DSM-IV). To assure the independence of multiplex subjects, we randomly selected only one affected sibling from each multiplex family. A total of 702 patients from these multiplex families were included in this study. The simplex families were recruited from another independent project [27]. They were interviewed by well-trained research assistants using the Diagnostic Interview for Genetic Studies. A total of 210 simplex families were recruited. Therefore, a total of 912 patients with schizophrenia were included in this genotyping study.

The normal controls were selected from a representative Taiwanese supernormal genomic sample [28] with inclusion criteria of age over 60 and Short Portable Mental Status Questionnaire score above 14. They were recruited by the Institute of Biomedical Science, Academia Sinica. A total of 600 supernormal control individuals (313 males and 287 females) were included in this study.

#### Subjects for replication

**Sample 1:** A total of 249 subjects were recruited from the inpatient facilities and day-care units of three psychiatric hospitals located in northern Taiwan; the Department of Health Taoyuan Mental Hospital, Bali Mental Hospital and Ju-Shan hospital. The enrolled patients had fulfilled the diagnostic criteria for schizophrenia as outlined by DSM-IV [29]. The diagnosis of schizophrenia was based on a review of all available medical records and confirmed by two board-certified psychiatrists. Patients with a concurrent diagnosis of a substance abuse disorder or with a clinically significant medical or neurological disease were excluded. The study subjects were recruited between November 1, 2006 and December 31, 2008.

**Sample 2:** A total of 139 subjects were recruited from the outpatient clinics of National Taiwan University Hospital. The patients, aged 18 to 65 years, met the DSM-IV criteria for schizophrenia. Subjects with mental retardation, schizoaffective disorders, bipolar affective disorder, organic mental disorders, and substance-related disorders were excluded. All the patients received blood withdrawn for DNA extraction and the examination of event-related potential (ERP) for the measurement of mismatch negativity (MMN). Detailed recruiting information of this sample has been published previously [30].

#### Subjects for the gene expression study

The subjects for the mRNA expression study were from the same project as those in the direct sequencing study. A total of 92 patients and 34 controls were included. The mean age of controls was 36.5 (± 10.3) years and 41.7% were male. The mean age of patients was 33.0 (± 9.1) years and 58.8% were male. There was no significant difference in age and gender of patients and controls. The mean age at onset of the patients was 20.2 (± 5.8) years and mean duration of illness was 12.8 (± 8.1). Most of them are chronic patients on regular antipsychotics.

#### Ethical statements

All the above subject recruiting projects were approved by the Institutional Review Boards of all the participating hospitals and institution, including National Taiwan University Hospital, Ju-Shan Psychiatric Hospital, the Institute of Biomedical Science, Academia Sinica, Department of Health Taoyuan Mental Hospital and Bali Mental Hospital. Written informed consent was obtained from all participants in these projects. The capacity for consent of these patients was assessed by their attending certified psychiatrists to rule out those participants whose psychotic symptoms or mentality were so severe that impaired their capacity for consent. All the psychiatric patients who were compulsory hospitalized did not allow entering these projects. All informed consents were obtained from patients themselves. Surrogate consent procedure was prohibited in these projects.

### DAO genomic sequencing regions defined by bioinformatics

#### Exon, splicing variant, and isoform analyses

The exon regions of DAO were evaluated not only for the 11 exons encoded in full-length cDNA, but also for transcripts and variants of expressed sequence tags. The exon data were processed by the Distributed Annotation System for the patterns of transcripts and variants located around the DAO genomic region.

The splicing variants and isoforms of DAO were processed by the Integrated Splicing Variants database [31]. Two isoforms (DAO mRNA transcripts of T1 and T2) and three expressed sequence tags variants were found in the DAO sequence region. The exons, splicing variants, and isoforms were compared with the Ensembl databases and were found to be consistent with them.

#### Promoter and the highly conserved regions of the introns

The promoter region was defined as the 2,000 base pairs before the start exon. The highly conserved regions were predicted by MultiPipMaker (http://pipmaker.bx.psu.edu/pipmaker/) output results [32, 33]. These conserved regions were arranged into clusters through percent identity plot (PIP) among humans, chimpanzees, mice, and rats by MultiPipmaker. The identity rate was defined by a similarity score divided by sequence length in a PCR product region. Regions were considered highly conserved if the identity was higher than 50% and the gaps among these regions were less than 10 bp.

**Fig 1:**
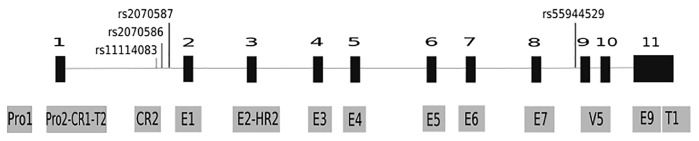
The sequenced regions of DAO in this study. DAO genetic sequencing regions (gray box) are displayed throughout the promoter (pro), and 11 exons (E) (black box). These sequenced regions include the transcripts (T) and variants (V) of expressed sequence tags, the highly conserved region (CR) and the previously significant disease-associated haplotype region (HR). The primer pairs for all sequencing regions are presented in Table A in S2 File. The SNPs of rs11114083, rs2070586, rs2070587, and rs55944529 genotyped in this study are indicated within the genetic region.

The genomic positions of previously significant disease-associated haplotype regions [4–6] and highly conserved regions were integrated. These regions were compared with exon regions and the splicing junctions were defined. The sequencing regions of primers were designed by Primer3 (http://frodo.wi.mit.edu/primer3/) [34] to avoid repeat sequencing in the same region. The sequencing regions including the promoters, 11 exons (including the transcripts and variants of expressed sequence tags, the highly conserved region and the previously significant disease-associated haplotype region [4–6] are shown in Fig 1.

### Direct sequencing

The genomic regions of DAO selected using the above bioinformatics procedures were amplified by polymerase chain reaction (PCR). PCR products were purified with exonuclease I and shrimp alkaline phosphatase (USB Corporation, Cleveland, OH, USA) and sequenced from both ends. DNA sequencing reactions were performed with BigDye Terminator Cycle Sequencing Version 3.1 (Applied Biosystems, Foster City, CA, USA) followed by analysis on an ABI 3730xl DNA Analyzer (Applied Biosystems, CA, USA).

#### Sequence alignment and analyses

The resulting sequences were compared and aligned using the Polyphred Sequence Alignment Editor (http://droog.gs.washington.edu/polyphred/). The reference sequences were obtained from the National Center for Biotechnology Information (NCBI) website (http://www.ncbi.nlm.nih.gov/).

### SNP genotyping

The selection criteria for large scale genotyping of genetic variants identified from direct sequencing were as follows: for less common variants (minor allele frequency in these subjects between 0 to 10%), > 2% difference in minor allele frequency between normal controls and the patients with schizophrenia; for common variants (minor allele frequency was between 10% to 50%), > 4% difference in minor allele frequency. All SNP genotyping was performed using the matrix-assisted laser desorption/ionization-time of flight mass spectrometry (MALDI-TOF MS) [35]. Primers and probes flanking the SNPs were designed using SpectroDESIGNER software (Sequenom, San Diego, CA, USA). A DNA fragment (100–300 bp) encompassing the SNP site was amplified by PCR (GeneAmp 9700 thermocycler, Applied Biosystems, CA, USA) according to the manufacturer’s instructions.

After removing the un-incorporated deoxynucleotide triphosphate (dNTP) and inactivating the shrimp alkaline phosphatase from the PCR product, primer extension was performed by adding the probe, Thermo Sequenase (Amersham Pharmacia, Piscataway, NJ, USA) and an appropriate dideoxynucleotide triphosphate (ddNTP/dNTP) mixture, followed by 55 cycles of denaturing at 94°C for 5 sec, annealing at 52°C for 5 sec, and extension at 72°C for 5 sec. The other extension products were differentiated by mass through MALDI-TOF [36].

### Real-time reverse transcriptase polymerase chain reaction (RT-PCR)

DAO expression was assessed using the lymphoblastoid cell lines transformed from lymphocytes by the Epstein-Barr virus (EBV). These transformed lymphoblastoid cells were washed once with 1x ice-cold phosphate buffered saline before total RNA extraction. Trizol Reagent (Invitrogen Life Technologies, Grand Island, NY, USA) was used according to the manufacturer’s guidelines to extract total RNA.

Real-time RT-PCR was performed for DAO and a housekeeping gene, TATA-box binding protein (TBP), using pre-designed gene-specific TaqMan® probes and primer sets (Hs01126151_m1 for DAO and Hs00920497_m1 for TBP) purchased from Applied Biosystems (Applied Biosystems, CA, USA). Real-time RT-PCR amplification was conducted using RevertAid™ H minus first strand cDNA synthesis kit (Fermentas, Waltham, MA) with random hexamer and real-time PCR on an ABI StepOne Plus System (Applied Biosystems, CA, USA), according to the manufacturer’s instructions. Gene expression was quantified relative to TBP expression using StepOne Software (Applied Biosystems, CA, USA) and the relative quantification method. The relative expression level of DAO compared with that of TBP was defined as CT = [CT_DAO_CT_TBP_], where CT was the cycle threshold. The DAO mRNA/TBP mRNA ratio was calculated from 2 ^CT^ × K, in which K was a constant.

### Neuropsychological assessment

#### CPT

A CPT machine from Sunrise System, v. 2.20 (Pembroke, MA, USA), was used to assess sustained attention. The procedure has satisfactory reliability, and has been described in detail elsewhere [16]. Briefly, numbers from 0 to 9 were randomly presented for 50 msec each, at a rate of one per second. Each subject undertook two CPT sessions: the undegraded 1–9 task and the 25% degraded 1–9 task. Subjects were asked to respond whenever the number “9” preceded by the number “1” appeared on the screen. A total of 331 trials, 34 (10%) of which were target stimuli, were presented over 5 minutes for each session. One signal-detection index of performance on the test, sensitivity (d´), was derived from the hit rate (probability of response to target trials) and false-alarm rate (probability of response to non-target trials) [37]. Sensitivity is defined as an individual’s ability to discriminate target stimuli from non-target stimuli.

In this study, the z-score of d’ on CPT was used as the endophenotype indicator for schizophrenia. The cut point of d’ was set as -2.5 from our previous family genetic study [17]. In this study, 298 patients had a z score of d’ on undegraded CPT below -2.5 (deficit) and 456 patients had a score above -2.5 (non-deficit); 374 patients had a z score of d’ on degraded CPT below -2.5 (deficit) and 358 patients had a score above-2.5 (non-deficit).

#### WCST

A computerized version of the WCST [38] used in a previous study of a Taiwanese population was used in this study. During the WCST, subjects were required to match response cards to the four stimulus cards along one of three dimensions (color, form, or number) by pressing one of the four number keys (1–4) on the computer keyboard. The testing in this study continued until all 128 cards had been sorted. In this study, the indices of WCST for association analyses were Perseverative Errors [39]. The indicator was found to be impaired in schizophrenic probands [18, 19] and in the first degree relatives of schizophrenic probands [20]. Based on the familial distributions of the z-score of Perseverative Errors, schizophrenic patients with a z score of Perseverative Errors ≥ 1 were defined as having deficit on the WCST. A total of 263 patients had a z score of Perseverative Errors above 1 (deficit) and 417 patients had a score below 1 (non-deficit).

#### Measurement of mismatch negativity (MMN)

The detail information about the measurement of MMN has been published previously [30]. Briefly, audiometry testing was used to exclude subjects who could not detect 40-dB sound pressure level tones at 500, 1000, and 6000 Hz presented to either ear. Subjects were seated in a comfortable recliner in a sound-attenuating and electrically shielded booth. The auditory stimuli were generated by a Neuroscan STIM system and were presented to subjects binaurally via foam insert earphones. The data was recorded by a Neuroscan ACQUIRE system (NeuroScan, Inc., El Paso, TX). The EEG signals were recorded with an electrode cap (Quik-Cap, NeuroScan, Inc., Charlotte, NC) from 32 scalp locations (10–20 system). An auditory oddball paradigm of duration MMN of approximately 30-min duration was given. The duration of standard stimulation and deviant stimulation were 50 msec and 100 msec, respectively. Stimuli occurred in a pseudorandom order with probability of occurrence 0.9 for standard tones and 0.1 for deviant tones. The MMN session was continued until a minimum of 225 artifact-free deviant trials had been collected online.

All the electroencephalographic data were processed using Neuroscan Edit 4.3 software (Compumedics USA, Charlotte, North Carolina). Semi-automated procedures using the Tool Command Language (TCL) batch processing language began with EOG artifact reduction through a built-in pattern-recognition algorithm. MMN waveforms were generated by subtracting the standard ERP from the deviant ERP. MMN indices were measured as the mean voltage from 135 to 205 milliseconds [40, 41].

### Statistical analysis

Hardy-Weinberg equilibrium was assessed by using the ALLELE procedure in SAS/GENETICS release 8.2 [42]. Haploview software was used to construct haplotype blocks with strong linkage disequilibrium for which the one-sided upper 95% confidence bound on D′ was >0.98 (that is, consistent with no historical recombination) and the lower bound was above 0.7 according to the criteria proposed by Gabriel et al. [43]. Haplotype frequencies were compared between the case and control groups by using the chi-square test or Fisher’s exact test where appropriate. The false discovery rate method [44] was used to correct for multiple comparisons. The relative expression levels of DAO between normal controls and schizophrenics were compared using a non-parametric Mann-Whitney U test for independent groups. A p-value less than 0.05 was considered significant. The MMN indices of the Fz electrode among patients carrying different number of the risk haplotype were compared using analysis of covariance (ANCOVA) after controlling age as a covariate.

## Results

### Association of single nucleotide polymorphism and schizophrenia

According to bioinformatics analyses (Fig 1), 15 amplicons were designed to search for the genetic variants of the DAO gene (Table A in S2 File). We found 13 SNPs (4 on the promoter region, 9 on the intronic region) through direct sequencing (Table B in S2 File). Five of the 14 SNPs fulfilled the criteria for further genotyping (Table 1). We found one novel SNP (ss73689521) located at intron 8 and reported this to the NCBI. This SNP (ss73689521 or rs55944529) showed a borderline significant association with schizophrenia (nominal p = 0.016, corrected p = 0.077) in the original sample (912 patients and 600 controls). The risk allele is the C allele and it affects the risk for schizophrenia with an odds ratio of 1.29 (95% confidence interval between 1.05 and 1.58). This SNP did not show a significant association in the replication sample due to its small sample size.

**Table 1:** Single locus association analysis of the five SNPs of DAO selected from the direct sequencing results in the original and replicated samples.

SNP information				Original sample (912 patients, 600 controls)				Replicated sample (Sample 1 + Sample 2) (388 patients)			Combined sample (1300 patients, 600 normal controls)		
SNP ID	Chromosome position^a^	Gene Location	Allele^b^	MAF^c^ Patient	MAF^c^ Control	Allele-wise p	Genotype-wise p	MAF^c^Patient	Allele-wise p	Genotype-wise p	MAF^c^ Patient	Allele-wise p	Genotype-wise p
rs2070585	107798175	5’UTR	G/A	0.049	0.056	0.40	0.69	-	-	-	-	-	-
rs11114083	107801621	Intron 1	C/A	0.381	0.359	0.23	0.20	0.384	0.25	0.52	0.382	0.18	0.13
rs2070586	107801849	Intron 1	G/A	0.370	0.356	0.42	0.44	0.391	0.11	0.10	0.377	0.21	0.21
rs2070587	107801872	Intron 1	T/G	0.370	0.356	0.41	0.50	0.383	0.22	0.18	0.374	0.21	0.28
rs55944529	107816559	Intron 8	C/T	0.133	0.165	0.016 (0.077) ^d^	0.026 (0.13) ^d^	0.170	0.78	0.84	0.144	0.09	0.22

^a^Chromosome: chromosome position version 36 at the National Center for Biotechnology Information (NCBI).

^**b**^The lower allele under slash is minor allele.

^c^MAF: minor allele frequency

^d^multiple test corrected p-value by False Discovery Rate.

**Fig 2:**
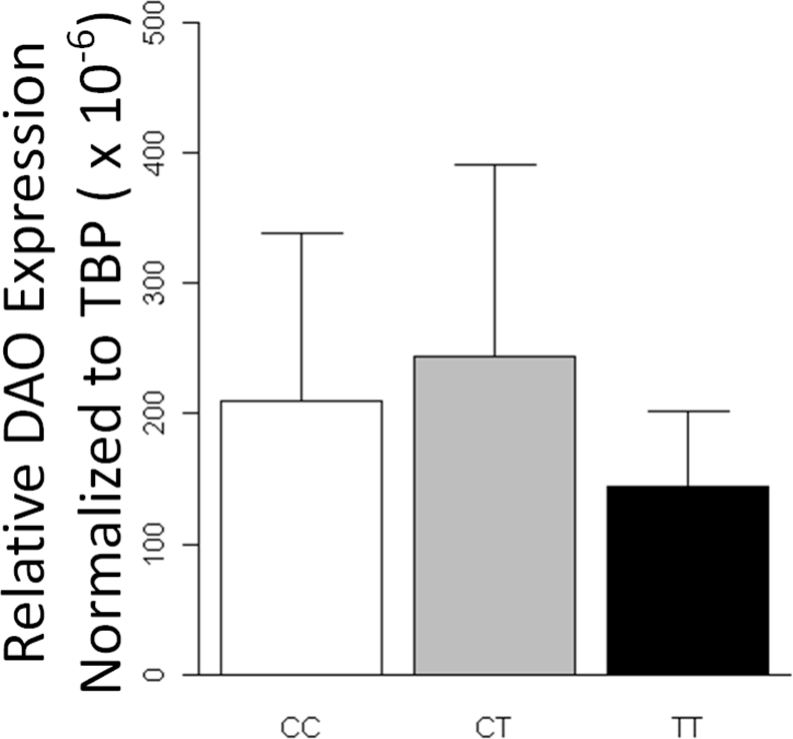
Differential DAO expression for genotypes of rs55944529. The transcriptional DAO expression is measured by real-time RT-PCR in the EBV-transformed lymphoblasts of 126 subjects. The relative DAO expressions for subjects with CC (n = 88) and CT (n = 26) genotypes, are significantly higher than that of TT genotype (n = 12) (Z = 2.17, df = 125, p = 0.029, using Wilcoxon two-sample test). Data are presented as mean ± SD.

By analyzing the differences in the relative expression of mRNA among the genotypes of the associated SNP rs55944529, we found a significantly higher level of DAO in the individuals carrying the risk allele (C allele, genotypes of CC and CT) compared to those without the risk allele (genotype of TT) (Z = -2.17, df = 125, p = 0.029) (Fig 2). However, there were no significant differences in the relative mRNA expression of DAO between patients and controls.

### DAO haplotype association with schizophrenia

A tetra-nucleotide (rs11114083- rs2070586-rs2070587-rs55944529) block was found in the patient group (Fig A in S2 File) using Haploview. Table 2 shows the results of the haplotype analysis of this haplotype block. We found highly significant differences in haplotype distribution between patient and control groups both in the original sample and in the replicated sample (global p < 0.0001 after multiple test correction). The haplotype frequencies of two haplotypes of AAGC and CGTC were significantly higher in the patient group in the original sample (corrected p < 0.0001 and p = 0.0089, respectively); however, the haplotype frequency of CGTT was not significantly different between patients and controls. The haplotype frequency of AAGC was also significantly higher in the patients in the replicated sample (n = 388) and in combined analysis (n = 1300) compared with the original controls (corrected p = 0.0003 and p < 0.0001, respectively) (Table 2); however, the haplotype frequency of CGTC was not significantly higher in the patients in the replicated sample, although significant in the combined analysis (corrected p = 0.047). There were no significant differences in DAO mRNA between these haplotypes (detailed data not shown).

**Table 2:** The tetra-nucleotide haplotype frequencies (HF) of the DAO gene and their associations with schizophrenia in the original and replicated samples.

	Original sample (912 patients, 600 controls)				Replicated sample (Sample 1 + Sample 2) (388 patients)			Combined sample (1300 patients, 600 controls)		
Haplotype^a^	Case (HF)	Control (HF)	Chi-square	P-value (corrected p^b^)	Case (HF)	Chi-square	P-value (corrected p^b^)	Case (HF)	Chi-square	P-value (corrected p^b^)
AAGC	0.37	0.294	18.18	<0.0001(<0.0001)	0.381	16.01	<0.0001(0.0003)	0.373	22.26	< 0.0001 (< 0.0001)
CGTC	0.49	0.435	8.94	0.0028 (0.0089)	0.447	0.24	0.63	0.478	5.93	0.0149 (0.0476)
CGTT	0.13	0.15	1.14	0.29	0.167	1.84	0.18	0.142	0.07	0.79
			**global p**	< 0.0001 (<0.0001)		**global p**	< 0.0001 (< 0.0001)		**global p**	< 0.0001 (< 0.0001)

Tetra-nucleotide haplotype: rs11114083—rs2070586—rs2070587—rs55944529 (intron 1- intron 1- intron 1- intron 8)

HF: haplotype frequency

All the degree of freedoms were equal to 1.

^a^The rare haplotypes were not listed here.

^b^The p value was calculated by Chi-square test and multiple test corrected by False Discovery Rate.

### Different haplotypes associated with different subgroups of schizophrenia

Table 3 shows that the CGTC haplotype of the tetra-nucleotide block (rs11114083- rs2070586-rs2070587-rs55944529) was specifically associated with the subgroups of schizophrenics with impairment in CPT and WCST. The corrected p values were significant in three deficient subgroups (p = 0.002–0.005), compared to those of the non-deficient subgroups (all were non-significant). We also observed that the AAGC haplotype was specifically associated with the subgroups without impairments in CPT and WCST. The corrected p-values were significant in three non-deficient subgroups (all p < 0.0001), compared to those of the deficient subgroups (p = 0.045–0.22). We did not assess CPT and WCST for the patients in the replicated sample, so that we cannot perform the analyses of the association between the haplotypes and the neurocognitive subgroups.

**Table 3:** Genetic associations of CGTC and AAGC tetra-nucleotide haplotypes of DAO in subgroups of schizophrenia with or without impairments in sustained attention and executive function.

Haplotype: rs11114083-rs2070586-rs2070587-rs55944529 (intron 1 -intron 1 -intron 1 -intron 8)			Case (HF)	Control (HF)	Chi-square^a^	P-value^b^ (Corrected p^c^)
CGTC	Deficient subgroup^d^	Undegraded CPT < -2.5 (298 cases)	0.521	0.435	11.72	0.0006 (0.002)
		Degraded CPT < -2.5 (374 cases)	0.509	0.435	9.93	0.0016 (0.0052)
		PER WCST ≥ 1 (263 cases)	0.519	0.435	10.20	0.0014 (0.0056)
	Non-deficient subgroup^e^	Undegraded CPT ≥ -2.5 (456 cases)	0.475	0.435	3.30	0.07
		Degraded CPT ≥ -2.5 (358 cases)	0.470	0.435	2.15	0.14
		PER WCST < 1 (417 cases)	0.477	0.435	3.48	0.06
AAGC	Deficient subgroup^d^	Undegraded CPT < -2.5 (298 cases)	0.347	0.294	5.19	0.023 (0.061)
		Degraded CPT < -2.5 (374 cases)	0.346	0.294	5.73	0.017 (0.045)
		PER WCST ≥ 1 (263 cases)	0.338	0.294	3.32	0.069
	Non-deficient subgroup^e^	Undegraded CPT ≥ -2.5 (456 cases)	0.379	0.294	16.80	<0.0001 (0.0001)
		Degraded CPT ≥ -2.5 (358 cases)	0.392	0.294	19.32	<0.0001 (<0.0001)
		PER WCST < 1 (417 cases)	0.389	0.294	19.99	<0.0001 (<0.0001)

CPT: continuous performance test WCST: Wisconsin card sorting test PER: perseverative error

^a^Degree of freedom = 1

^b^The p value was calculated by Chi-square test

^c^Multiple test corrected by False Discovery Rate

^d^deficient subgroup was defined by z score of d’ < -2.5 on undegraded CPT or on degraded CPT, or by z score of perseverative error ≥ 1 on WCST

^e^Non-deficient subgroup was defined by z score of d’ ≥ -2.5 on undegraded CPT or on degraded CPT, or by z score of perseverative error < 1 on WCST.

### Association between haplotypes and MMN

We found that the AAGC haplotype of the tetra-nucleotide block (rs11114083- rs2070586-rs2070587-rs55944529) was associated with the MMN index. Table 4 shows that the more AAGC haplotype the patients carry, the poorer MMN they have (the more negativity means better MMN) after controlling for age.

**Table 4:** The MMN indices of Fz electrode of patients carrying different number of AAGC haplotype of the tetra-nucleotide block (rs11114083- rs2070586-rs2070587-rs55944529).

	Number of AAGC haplotype		
	0 (N = 53)	1 (N = 59)	2 (N = 23)
MMN index of Fz electrode (mean ± s.d.)*	-0.58 ±0.76	-0.31 ± 0.62	-0.17 ± 0.54

*: p = 0.037 for the additive model, p = 0.016 for the dominant model using ANCOVA after controlling for age.

## Discussion

By direct sequencing, we found a novel SNP rs55944529 located at intron 8 of the DAO gene. The frequency of the C allele of this SNP was significantly higher in the original patient sample than the controls, but not different in the replicated sample. The C allele of this SNP was associated with a higher level of mRNA expression of DAO in the EBV-transformed lymphocytes. The haplotype distribution of rs11114083- rs2070586-rs2070587-rs55944529 was significantly different between schizophrenia and control groups, and the haplotype frequencies of AAGC were significantly higher in the schizophrenia group, both in the original and replicated samples. The haplotype of CGTC was specifically associated with the subgroup of schizophrenia with deficits in sustained attention and executive function, while the haplotype of AAGC was specifically associated with the subgroup of schizophrenia without deficits. The haplotype of AAGC was associated with the poorer MMN index of ERP in schizophrenia patients.

The SNPs of DAO previously reported to be associated with schizophrenia are DAO-M4 (rs2111902 at intron 1), DAO-M5 (rs3918346 at intron 3), and DAO-M6 (rs3741775 at intron 4) in Caucasian populations [4–6]; however, this result was not replicated in Chinese [7] and Taiwanese populations [8]. In this study, we found a novel SNP rs55944529, located at intron 8. Although the novel SNP rs55944529 had a low significant level of association in the original sample and not significant in the replicated sample, the haplotypes composed of this novel SNP (rs55944529) and the other three SNPs in the public databank (rs11114083, rs2070586, and rs2070587) did show a highly significant association both in the original and replicated sample. This highlights the importance of exploring novel SNPs from a specific population sample by direct sequencing, rather than by using SNPs only from the public databank. It also suggests that the regions of DAO associated with schizophrenia might be different in different ethnic groups.

The most recent report from the Psychiatric Genomics Consortium (PGC) didn’t reveal association between DAO gene and schizophrenia (odds ratio = 0.979, p = 0.65 for the SNP rs55944529 in CEU sample (Sweden 1–6)) [45]. The genetic analyses of major histocompatibility region in Han Chinese in Taiwan and Caucasians revealed Taiwan’s Han Chinese differ drastically in genotypic information compared with Caucasians but are relatively homogeneous in itself [46]. The different population structure may explain the differences between the results of the PGC and our study. The genome-wide association study (GWAS) in Japan, using 575 patients and 564 controls, didn’t reveal significant association evidence of DAO with schizophrenia [47]. The GWAS study in China, using 498 patients and 2025 controls from the Han Chinese population, revealed borderline significance (p = 0.06) for the SNP rs3918347 [48], which is 890 bp from our SNP rs55944529. They didn’t perform haplotype analyses in their studies and it is still unclear whether the specific associated haplotype in our study is replicated in these different samples. We suggest that the SNP rs55944529 is not the real associated genetic variant, but the underlying genetic structure captured by the AAGC haplotype is associated with schizophrenia.

The most consistent result of this study is the association evidence of AAGC haplotype with schizophrenia. It has been reported that the MMN index is associated with NMDA receptor function [49]. The poorer MMN in schizophrenia patients is consistent with the evidence of hypofunction of NMDA receptor function in schizophrenia [50, 51]. The association between the AAGC haplotype and poorer MMN implies that the haplotype AAGC might have negative impact upon DAO activity (maybe elevation), next lower the function of NMDA receptor, then influence MMN index, and at last elevate the risk of schizophrenia. However, we failed to find the association of mRNA expression with this haplotype.

The genetic information captured by the haplotypes is much more than a single SNP. The genetic mechanism underlying the haplotypes may be complex and multiple true associated genetic variants may lie in the haplotype region. We found that the haplotype of CGTC was specifically associated with the deficient subgroup, and the haplotype of AAGC with the non-deficient subgroup. It implies there may be some SNPs in the haplotype region, influencing the biological function of DAO, then having impact upon the neurocognitive function. Because the rs55944529 is not the true functional genetic variant from our association results, we suggest that the association of rs55944529 with the mRNA expression of DAO may be through the association with the neighbor functional SNPs, which may have impact upon mRNA expression.

The mechanism underlying the genetic association of DAO with schizophrenia remains unclear. Several studies have reported increased DAO expression and activity in the postmortem brain of schizophrenic patients [52–56]. Our novel finding reveals that the genotypes of a SNP rs55944529 at intron 8 have effects on the mRNA expression of DAO. With regard to the association between genotypes and other endophenotypes of schizophrenia, one study found that an SNP in the 5’-UTR in DAO (rs4623951) was associated with sensorimotor gating, working memory, and personality patterns in healthy males in a Greek population [57]. A functional imaging study revealed that the DAO genotypes (rs3918346 at intron 3) were related to the degree of deactivation in the left precuneus and greater activation in the right posterior cingulate gyrus, reflecting a reallocation of cognitive resources [58]. One study found no association of three DAO SNPs (rs3918346 at intron 3; rs3741775 at intron 4 and rs3918347 at intron 10) with performance on a broad range of cognitive tasks, including CPT and WCST [59], while our study found a tetra-nucleotide haplotype across intron 3 and intron 8 to be associated with neurocognitive function in schizophrenia assessed by CPT and WCST. The various SNP coverages, cognitive tests, and ethnicities across these studies may explain the diversity of these results.

There are some limitations to our study. First, we did not measure the plasma D-serine level and DAO enzyme activity in the peripheral blood cells, which may relate to the DAO functional pathway more directly than the level of DAO expression in the EBV-transformed lymphocytes we measured in this study. Second, we did not have CPT and WCST data in the replication sample for analysis. Third, though we found the risk AAGC haplotype was associated with poorer MMN, we failed to find the association of this haplotype with the mRNA expression of DAO.

## Conclusion

In summary, the most consistent finding of our study was the evidence for an association between the haplotypes of DAO and schizophrenia. Our findings suggest that the DAO gene is a susceptibility gene for schizophrenia and the true functional genetic variants related to this disorder may lie on the genomic region between intron 1 and intron 8.

## Supporting Information

S1 File Genotyping raw data of the original sample. (CSV)

S2 File Table A, Table B, Fig A.
(DOC)
