# Supplementary material for: Haplotypes of the D-Amino Acid Oxidase Gene Are Significantly Associated with Schizophrenia and Its Neurocognitive Deficits
Source: PLoS One. 2016 Mar 17;11(3):e0150435. doi: 10.1371/journal.pone.0150435 (PMC4795637; doi:10.1371/journal.pone.0150435)

**S2 File**

**Table A.** The primer pairs for polymerase chain reaction in sequencing study of DAO genetic exons (E), promoter (Pro), transcripts (T), variants of expressed sequence tags (V), the highly conserve region (CR) and the previously significant disease-associated haplotype region (HR).

| **Transcript ID** | **Primer F** | **Primer R** | **PCR Sizes (bps)** |
| --- | --- | --- | --- |
| DAO_Pro1 | AGAATTGGTACACCGCAATTT | caagcaatccttctgcttca | 447 |
| DAO_Pro2 | agtgtgcagtggtgccatc | GGGTCAGCTTTAGCAATCCA | 383 |
| DAO_CR1 | AGGTGATCCACCCACCAT | CCTGTGGACAGGCCTTGG | 700 |
| DAO_T2 | CCATGGAACGTCCTGACC | TCACGCTGTAAGAGGGGAGT | 680 |
| DAO_CR2 | GCAGATGAAGAGCTTGTGTCT | GAGGTGATGTGACTTACCTGAAG | 700 |
| DAO_E1 | GACGCTCCCCAGAGAAAGTT | GCCCAGGCTGATCTGGAA | 686 |
| DAO_E2 | GTGGCTCATAATCCCCAAAG | GCCATGCTAATCAAAGGCTA | 699 |
| DAO_HR2 | TGCTGTTAAATTGGCAGAGG | CTCTTGGTCCCATGAAAGGT | 585 |
| DAO_E3 | GGCACAATTTCGGCTTACTG | GGGTGTGGGGTCATACAGAC | 730 |
| DAO_E4 | CTAAGGTCACACAGCCAAGTG | CATTAGGGATTTGAAAGAGGTTG | 700 |
| DAO_E5 | CTCTGAATCTCATCGCCTTTG | GCCAGCTCTCTAGACCCAAAA | 696 |
| DAO_E6 | ATTTCCCCCATCCAAAGAAC | ATCTCGCGCTTGGTAAACAC | 726 |
| DAO_E7 | AGCTGTGAACTCAGCTCCAA | GTGGTGGGCACCTGTAATCT | 736 |
| DAO_V5 | GATGTTAATACTGTAGCAGAGCTGAC | ATGGCCAGACAGGATTCAT | 700 |
| DAO_E9 | TGATGAGGATGAGTTGGTACTGA | CCACTCCCAGTCACACCATT | 700 |
| DAO_T1 | AGGTGGCATCTGGCTTTG | GCCAGATAGGTTTAGCAAGCA | 697 |

**Table B.** SNPs obtained from direct sequencing.

| NGC Primer_ID | Genetic Region | Chromosome Pos 36 | SNP ID | Chromosome Pos 37 | Location |
| --- | --- | --- | --- | --- | --- |
| 13806 | DAO.CR1 | 107775833 | rs11114081 | 109273367 | Promoter |
| 13811 | DAO.CR1 | 107775885 | rs113967217 | 109273419 | Promoter |
| 13802 | DAO.T2 | 107776244 | rs113736364 | 109273778 | Promoter |
| 13812 | DAO.T2 | 107776247 | rs111434958 | 109273781 | Promoter |
| 13830 | DAO.T2 | 107776512 | rs2070585 | 109274046 | intron1 |
| 13804 | DAO.CR2 | 107779958 | rs11114083 | 109277492 | intron1 |
| 13807 | DAO.CR2 | 107780186 | rs2070586 | 109277720 | intron1 |
| 13799 | DAO.CR2 | 107780209 | rs2070587 | 109277743 | intron1 |
| 13803 | DAO.E2 | 107783944 | rs2302882 | 109281478 | intron3 |
| 13932 | DAO.E3 | 107786038 | rs3741776 | 109283572 | intron4 |
| 13929 | DAO.E5 | 107789376 | rs17041050 | 109286910 | intron5 |
| 13925 | DAO.E4 | 107786552 | rs3741774 | 109284086 | intron5 |
| 14190 | DAO.V5 | 107794896 | rs55944529 | 109292430 | intron7 |

Chromosome Pos 36: chromosome position version 36 at the National Center for Biotechnology Information (NCBI).

Chromosome Pos 37: chromosome position version 37 at the NCBI.

**Fig A. The haplotype structure defined by D’ in the combined sample of 1300 patients**


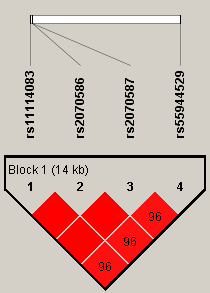


The r^2^


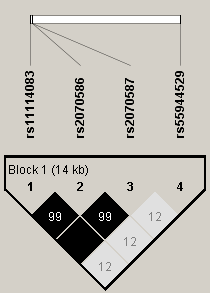

Supplement: S2 File — Table A, Table B, Fig A. (DOC) [file pone.0150435.s002.doc]
